# Biocompatible memristive device based on an agarose@gold nanoparticle-nanocomposite layer obtained from nature for neuromorphic computing

**DOI:** 10.1038/s41598-023-32860-6

**Published:** 2023-04-20

**Authors:** Youngjin Kim, Jun Seop An, Donghee Lee, Seong Yeon Ryu, Yoon-Chul Hwang, Dae Hun Kim, Tae Whan Kim

**Affiliations:** 1 Research Institute of Industrial Science, Hanyang University, Seoul, 04763 Republic of Korea; 2 Department of Electronic Engineering, Hanyang University, Seoul, 04763 Republic of Korea

**Keywords:** Electrical and electronic engineering, Materials for devices, Nanoscale materials

## Abstract

Natural, organic, materials-based artificial synaptic devices have been in the spotlight for wearable/flexible devices due to their lightweight, biocompatibility, and scalability. In this study, an electronic memristive device based on agarose extracted from plants in the Rhodophyceae class was fabricated, and its memory characteristics and analog data processing capabilities were evaluated. The Al/agarose@gold nanoparticle (AuNP) film/indium-tin-oxide (ITO)-structured memristive device exhibited reliable resistive switching characteristics with excellent retention with a large Ron/Roff ratio of 10^4^. Also, analog conductance changes in our device were achieved with power consumption at the pJ level. This notable behavior could be maintained under mechanical deformations from a flat to a 4-mm bent state. In the recognition simulation based on the device's performance, an 91% accuracy and clear digit classification were achieved.

## Introduction

The structure of a conventional computer, the von Neumann structure, consists of a central processing unit (CPU) and a memory that stores information^1–3^. However, the von Neumann structure has a problem of excessive energy consumption and time delay in signal transmission due to the bottleneck between the CPU and the memory. Accordingly, a new concept for a computing system with high energy efficiency and excellent performance that overcomes the problems of existing computer system is needed^4^. A neuromorphic system that mimics the neuron-synaptic structure of a biological nervous system is proposed as an alternative to the existing von Neumann structure^5–9^. The human brain consumes less energy and processes large amounts of data in parallel^10^. The reason is that 10^11^ neurons and 10^4^ synapses between neurons are involved when the brain processes information^11^. Moreover, the most important part of a neuromorphic system is the implementation of a synaptic device that simultaneously handles signal processing, learning, and memory^12,13^. Synapses in biological systems are responsible for learning and for remembering the signal transmitted by neurons through a change in the synaptic weight. This change in synaptic weight corresponds to the characteristic of storing information as with the resistive switching in a memristor^14–17^.

Recently, some environmentally friendly materials, such as silk, chitosan, collagen, and albumen, have been explored as active building blocks in memristive devices^18–23^, and these environmentally friendly material-based memristive devices have been demonstrated to be promising candidates for replacing devices that use silicon-based materials^24,25^. Especially, memristive devices based on environmentally friendly materials, due to their being human friendly and having biodegradable properties, have received great attention for applications in wearable and implantable devices^18–23^. Agarose can be obtained from certain species of red seaweeds belonging to the Rhodophyceae class^26,27^. It is a suitable biomaterial for use as a core component in bio-compatible memristive devices. It has excellent mechanical properties, including excellent thermal stability at temperatures up to about 250 °C, high surface stiffness, and good flexibility, as well as excellent transparency due to close chain packing and extensive inter-inter molecular hydrogen bonding between hydroxy groups^26,27^, and its practical applications can be widened by using functional-group modification. The biocompatible, bioinert, and biodegradable properties of agarose have already been proven in many bio-field studies and bio-applications, such as drug delivery, cancer treatment, tissue regeneration including heart/wound/skin, and attachment to brain and cornea^28–31^. Finally, its macromolecular networks caused by high cross-linking and the self-assembly of hydrogen bonds allow sufficient diffusion and transport to take place in the active layer of the memristive device.

In this manuscript, we report a biocompatible memristive device based on a composite film consisting of agarose@gold nanoparticles (AuNPs) as an active layer. AuNPs are used as additives due to their chemical stability, high work function, and nontoxicity^32^. Especially, the superior electron trapping properties of AuNPs enable to obtain good resistance-switching characteristics and maintain the resistance state^33^. The data storage capability of this memristive device was evaluated, and its resistance switching mechanism was demonstrated. We investigated the feasibility of using this memristive device in flexible electronics. The analog data processing capabilities of the agarose@AuNP composite-based device were demonstrated. Then, we investigated the recognition capability of our memristive device, which is based on the device’s behavior via a synaptic spike. We believe that the results of this research on agarose-based memristive devices provides a valuable step in the realization of eco-friendly, flexible, and wearable electronics.

## Results and discussion

### Structural investigation of Agarose@AuNPs-based nanocomposite device

**Figure 1:**
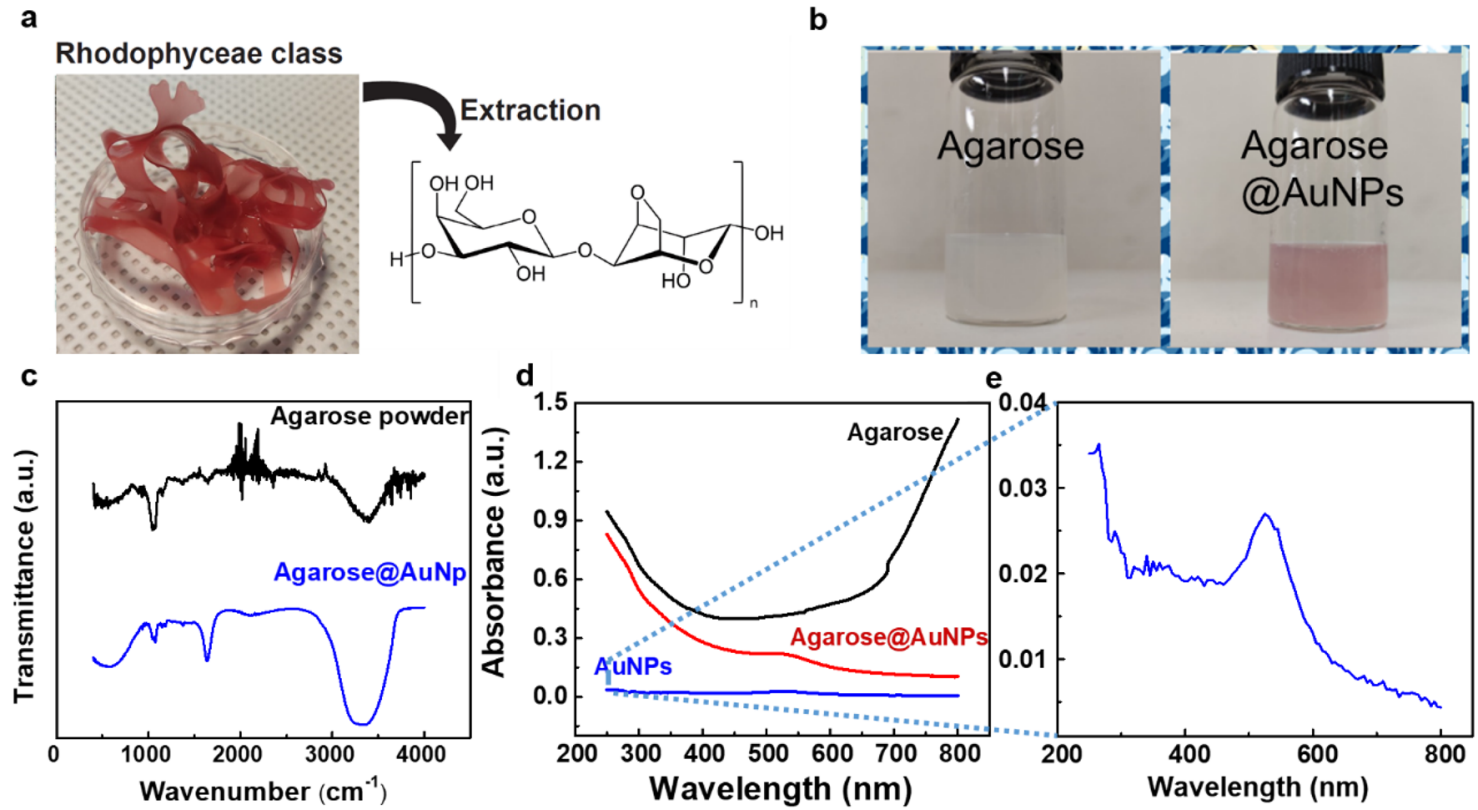
(**a**) Photograph of a plant in the rhodophyceae class and the molecular structure of agarose. (**b**) Photographs of the agarose and the agarose@AuNP solutions. (**c**) FTIR results for the agarose powder and for an agarose@AuNP composite film. (**d**) UV–vis spectra for agarose, AuNPs, and agarose@AuNPs. (**e**) Enlarged UV–vis spectrum for AuNPs.

Agarose consists of a linear polysaccharide that is composed of 1,4-linked 3–6-anhydro-l-galactose and 1,3-linked β-d-galactose units, as shown in Fig. 1a. The photographs in Fig. 1b show the agarose solution (left) and the agarose@AuNP solution (right). After 1 wt.% of AuNPs had been introduced into the agarose-based solution, the resulting aqueous solution was purple due to the dispersed gold nanoparticles. The distinctive absorption result of agarose powder in Fourier-transform infrared spectroscopy (FT-IR) can be seen at 1050 (the glycosidic bonds), 1175 (other C–O–C bonds), 1600 (C=C bonds), and 3400 cm^-1^ (O–H bonds)^34^, as shown in the upper part of Fig. 1c. After the introduction of the AuNPs, the distinctive peaks are still maintained (bottom of Fig. 1c); i.e., the basic molecular structure of agarose was maintained after the introduction of the AuNPs. Compared to the result for the agarose powder, however, the peak intensities of the agarose@AuNP composite film were changed due to high-crosslinking and self-assembling of the hydrogen bonds due to the water solvent^28^. Figure 1d shows the UV–vis absorption spectra for pure agarose, AuNPs, and an agarose@AuNP composite film. In the spectrum for the agarose@AuNP composite film, through a comparison with the absorption peak for pure agarose, we were able to attribute the distinguishing peak observed in the region of 534—543 nm to a surface plasmonic resonance effect caused by the AuNPs^35^ (Fig. 1e). This means that the AuNPs had been successfully mixed into the agarose polymer matrix.

**Figure 2:**
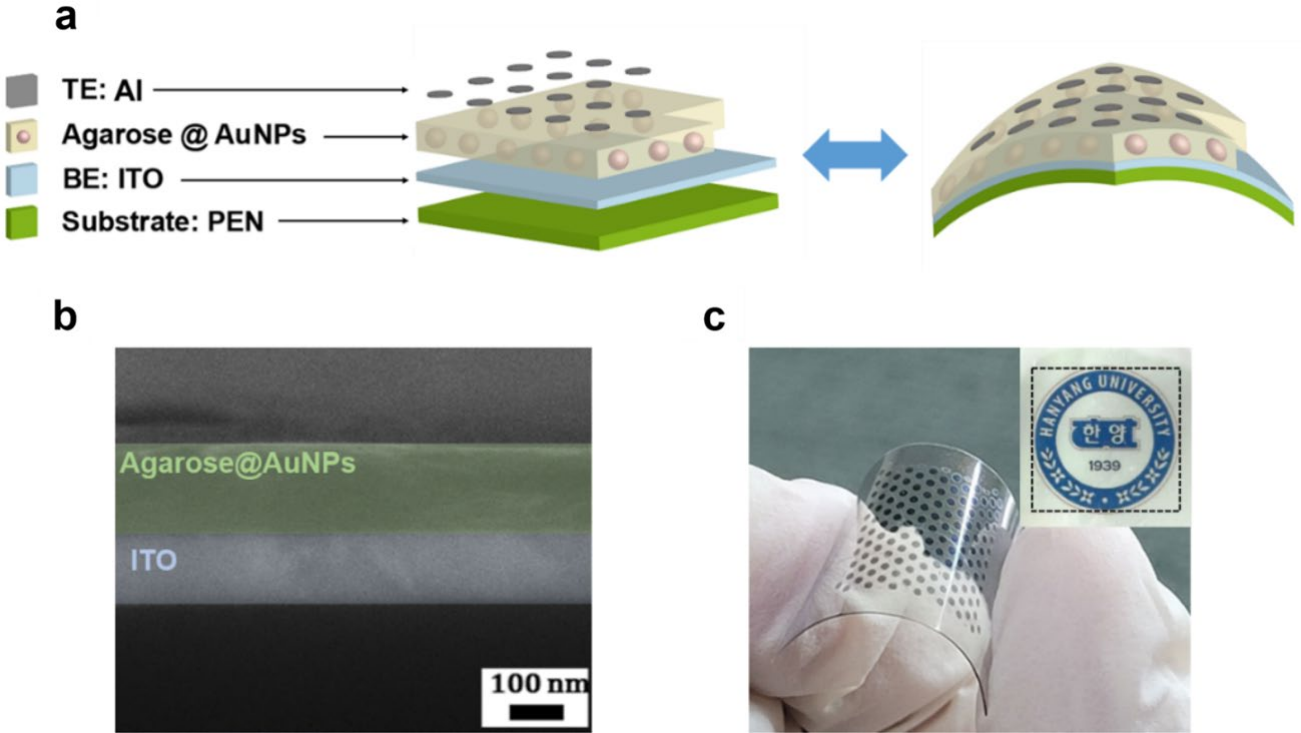
(**a**) Illustration of the Al/agarose@AuNPs/ITO-PEN structure in the device, and (**b**) cross-sectional SEM image of the agarose@AuNPs/ITO-structured film. (**c**) Photograph of the Al/agarose@AuNPs/ITO-PEN flexible memristive device. The inset is an image of the memristive device placed on top of the logo of Hanyang University.

A schematic illustration of the memory device fabricated with an Al/agarose@AuNP composite layer/ITO-PEN structure is shown in Fig. 2a. Figure 2b demonstrates that a 150-nm-thick active composite layer had been deposited between the two electrodes. Figure 2c shows a real photograph of the Al/agarose@AuNP composite film/ITO-PEN device to demonstrate its flexibility. Also, the agarose@AuNP composite film deposited on the ITO-PEN substrate was confirmed to be transparent, as shown in the inset of Fig. 2c.

### Resistance switching characteristics

**Figure 3:**
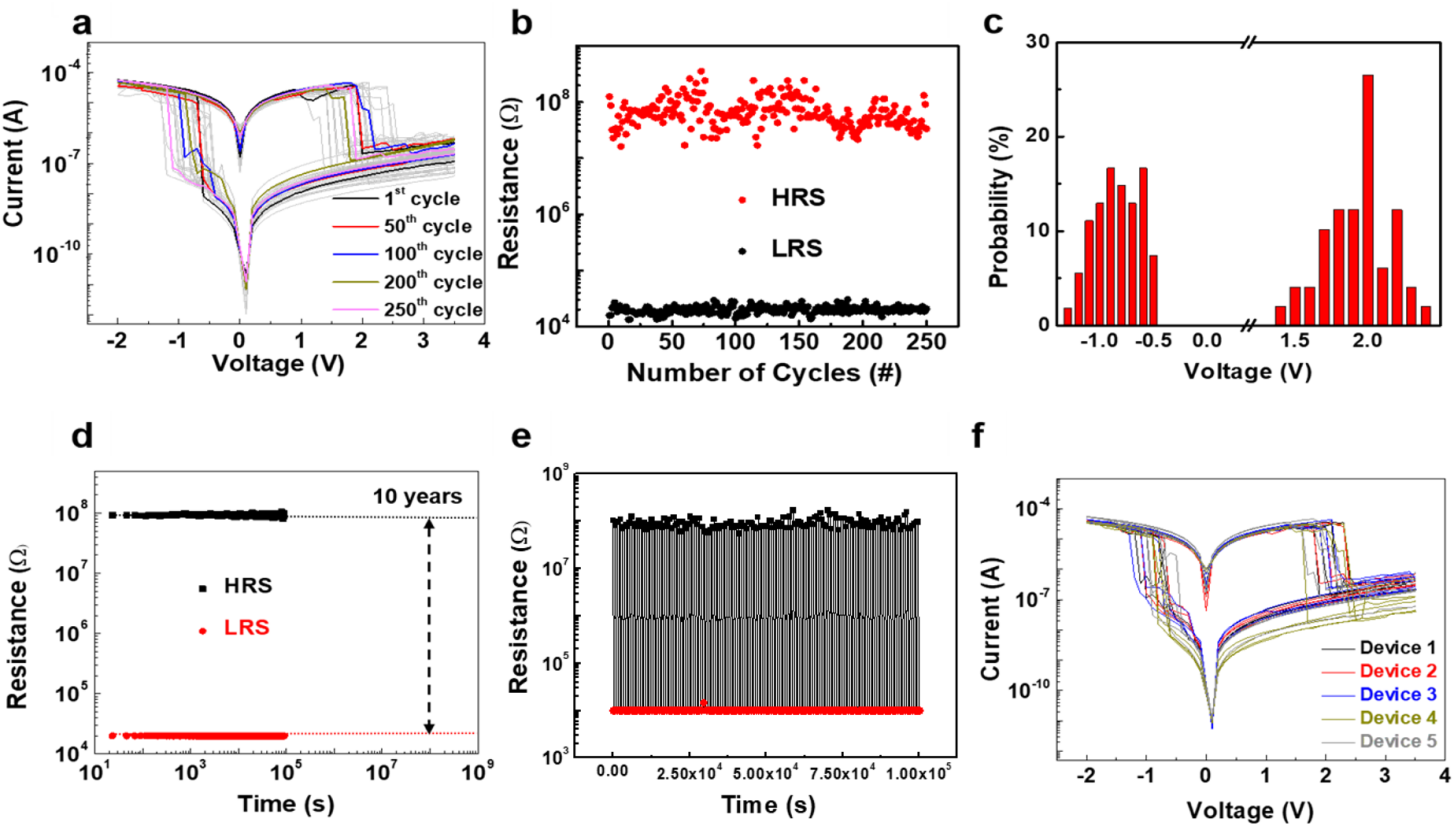
(**a**) *I–V* characteristics of the Al/agarose@AuNPs/ITO-PEN memristive device for 250 resistance switching cycles. (**b**) Endurance results and (**c**) probability distributions of the SET and the RESET operating voltages for the memristive device. (**d**) Retention results for the memristive device at 80 °C over a period of 10^5^ s at a read voltage of 0.5 V. (**e**) Endurance results in the pulse mode for 10^5^ cycles. The SET and RESET pulse heights (duration) of -1.5 V (1 ms) and 2.85 V (1 ms), respectively. (**f**) Device-to-device results for the Al/agarose@AuNPs/ITO-PEN memristive device. This test was conducted by randomly selecting four cells each from five devices.

The data storage capability of the Al/agarose@AuNP composite film/ITO memristive device was electrically characterized by using a voltage sweep (0 → −2 → 3.5 → 0 V) under a compliance current of 0.1 A. The biocompatible memristive device showed typical bipolar resistance switching behavior, as shown in Fig. 3a. When a negative external bias was applied to the memristive device, a resistance change was observed. The resistance of the device changed from a high resistance state (HRS, about 67.1 MΩ) to a low resistance state (LRS, about 22 kΩ) at −0.7 V, which is called the SET voltage. Subsequently, when the voltage was swept to the positive voltage region, the resistance switched from the LRS to the HRS (RESET process). Interestingly, a non-zero-crossing hysteresis is observed, which is presumed to be due to a capacitive effect caused by the large number of functional groups in biomaterials; moreover, the capacitor-like structure induces a residual current in the active layer^25,36^. After the first resistance switching process, 249 additional cycles of resistance switching were conducted. All the shapes for the trend of the current during resistance switching corresponding to a voltage sweep were very similar. Figure 3b shows the endurance results for 250 repetitive resistance switching operations. At a read voltage of 0.5 V volts, the average resistances and their standard deviations for the LRS and the HRS were 20.01 ± 3.05 kΩ and 74.87 ± 36.91 MΩ, respectively. The probability distributions for the SET and the RESET voltages for cycle-to-cycle repetitive switching are shown in Fig. 3c, and the average values are -0.84 ± 0.21 V and 1.95 ± 0.25 V, respectively. These switching results demonstrate that the Al/agarose@AuNP composite film/ITO memristive device can be applied as a reliable non-volatile memory. Next, the retention of the input data in the memristive device was investigated at a 0.1-V read voltage for 10^5^ s under a high temperature of 80 °C, and the result is shown in Fig. 3d. The resistance values exhibited minimal changes, and the input data stored in the memristive device are anticipated to be retained for over ten years on the basis of an extrapolation. In addition, our composite-based memristive device exhibited reproducible resistance-switching operation with a large R_on_/R_off_ ratio of 10^4^ for up to ~ 10^5^ switching cycles without any failure in the programmed pulse switching test [the SET and the RESET pulse heights (duration) were −1.5 V (1 ms) and 2.85 V (1 ms), respectively.] (Fig. 3e). To demonstrate the contribution of the AuNPs to this notable resistance switching in the Al/agarose@AuNP composite film/ITO memristive device, we prepared an Al/agarose/ITO memristive device as a control. Resistance switching in the control without Ag NPs under the same *I-V* sweep (0 → −10 → 10 → 0 V) was barely observed for 10 devices, as shown in Fig. . S1. Thus, we conclude that the AuNPs in the composite-based active film played a very important role in inducing resistance switching. Figure 3f shows the *I–V* sweep results for four randomly selected cells from each of five devices. The resistance switching parameters, including operating voltages and both resistance states, were almost the same as those in the single cell test. The average SET and RESET voltages from the device-to-device test were −0.88 ± 0.15 V and 2.10 ± 0.22 V, respectively.

### Resistance switching mechanism

**Figure 4:**
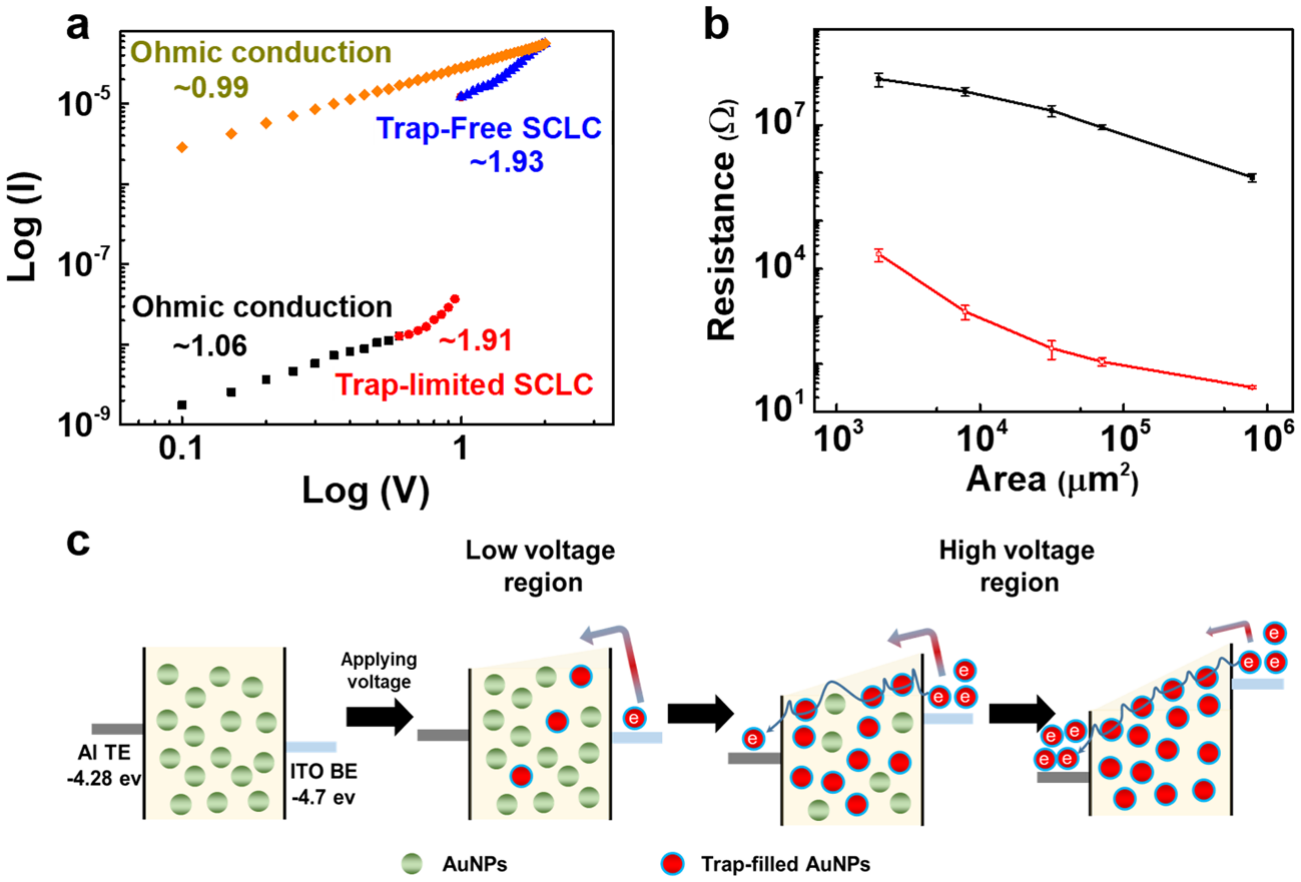
(**a**) Double-logarithmic *I–V* plot in the negative sweep region of 0 V → −2.0 V → 0 V) from the HRS to the LRS of the Al/agarose@AuNPs/ITO memristive device. (**b**) Electrode-area dependence of the memristive device, with the TE diameters ranging from 50 μm to 1 mm. Each resistance was collected at 0.5 V of read voltage. (**c**) Schematic illustration of the resistance switching mechanism in the memristive device.

To identify the resistance switching mechanism of the Al/agarose@AuNP composite film/ITO memristive device, we plotted the measured *I–V* characteristics on a log–log scale (Fig. 4a). In the low voltage region, a linear fitting (slope of 1.06) to the *I–V* curve pointed to an Ohmic conduction behavior (0 to 0.55 V). However, in the voltage range between 0.60 V and 1.00 V, the log–log curves appear to follow a quadratic function with a higher slope (1.91), pointing to a trap-limited space-charge-limited current (TCLC) behavior. After the resistance state had changed from the HRS to the LRS at 1 V, the fitting result showed a trap-free space-charge-limited current (trap-free SCLC) behavior with a slope of 1.93. After that, the ON state clearly showed an Ohmic conducting behavior with a slope of 1.06, which could be attributed to the small electric field between the two electrodes in this region. In Ohmic conduction, the number of trapped carriers is less than the number of thermally generated carriers; in such a case, Ohmic behavior, as described below, is detected, rather than the SCLC behavior induced by trapped carriers^14,37,38^:

1 $$ J = qn\mu \frac{V}{d} \left( { J \propto V } \right), $$

after which the gold nanoparticles inside the agarose further increased the number of trap sites so that a trap level was present near the Fermi level, and TCLC behavior was caused by the restricted movement of electrons (Mott-Gurney law)^14,37,38^:

2 $$ J = \alpha V + \beta V^{2} , \;when\; \alpha = \frac{nq\mu }{d};\;\; \beta = \frac{{9\mu \theta \varepsilon_{r} \varepsilon_{0} }}{{8d^{3} }};\;\;\; \theta = \frac{{N_{c} }}{{g_{n} N_{t} }}{\text{exp}}\left( {\frac{{E_{t} - E_{c} }}{{K_{B} T}}} \right) $$

Then, as the voltage was increased beyond the SET voltage, the number of free carriers within the active layer increased so that all traps were filled, the effects of the traps disappeared, and the current was found to have a trap-free SCLC behavior (Eq. 2). In this study, a sequential Ohmic region (n = 1), a Mott-Gurney law-governed region (n = 2), and a region of steep current increase at higher voltages were shown to exist^14,37,38^. In Eqs. (1) and (2), the symbols have the usual meanings: *J* is the current density, *q* the electronic charge, *n* the density of thermally generated charge carriers, $\mu$ the electron mobility (assumed to be independent of the charge-carrier density and the electric field), *d* the film’s thickness, $\varepsilon_{0} \varepsilon_{r}$ the permittivity with $\varepsilon_{0}$ being the permittivity of free space and $\varepsilon_{r}$ the dielectric constant of the material, $g_{n}$ the degeneracy of energy states in the conduction band, $N_{c}$ the effective density of states in the conduction band, $N_{t}$ the total trap density, $E_{t}$ the trap energy level, $E_{c}$ the energy at the conduction band edge, and $K_{B}$ and *T* the Boltzmann constant and the temperature, respectively. Therefore, the conduction mechanism is believed to be in accord with an SCLC mechanism, with the AuNPs acting as trap centers in the agarose@AuNP composite film. The electrode-area dependence of the resistance in our device was investigated, and the results were used to explain the device’s resistance switching mechanism. Significant changes in both resistance states were observed as the area of the electrode was varied (Fig. 4b), which means that the resistance switching, instead being due to a filamentary-based switching mechanism, takes place across the entire area of the electrode^39,40^. Therefore, the operating current can be further reduced by downsizing the device. On the basis of the above results, the resistance switching mechanism in our Al/agarose@AuNP composite film/ITO memristive device is illustrated in Fig. 4c.

### Artificial synaptic behavior

**Figure 5:**
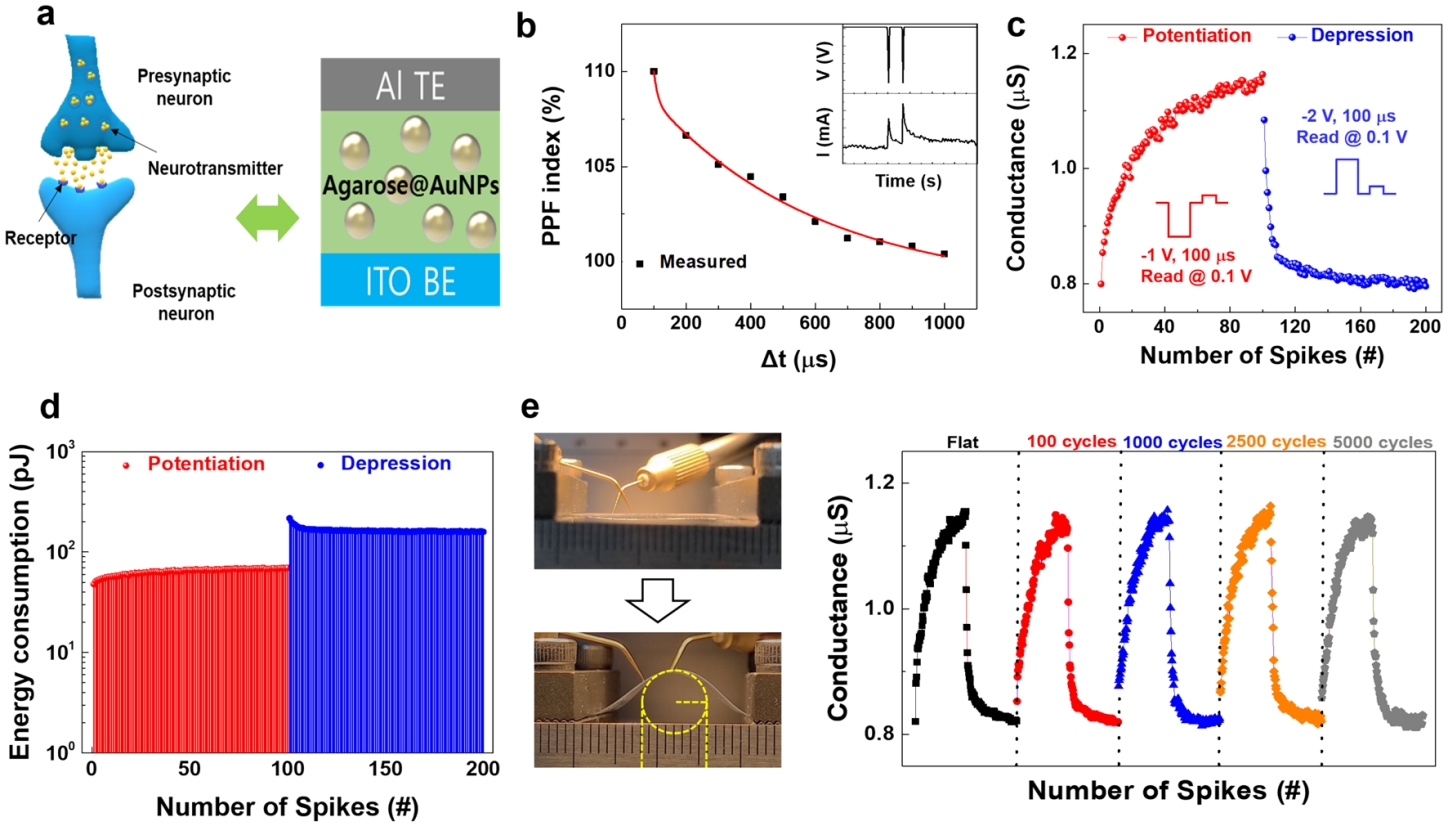
(**a**) Schematic diagram for biological signal transmission in a biological synapse, and comparison to an Al/agarose@AuNPs/ITO memristive device. (**b**) Correlation between the PPF index and the time interval. The inset image is the conductance change triggered by a paired pulse. (**c**) Synaptic LTP and LTD results, stimulated by consecutive electrical spikes. (**d**) The energy consumption on the LTP and LTD processes. (**e**) Photographs of the agarose@AuNP film-based device subjected to bending from the flat, and LTP and LTD characteristics of the device up to 5000 bending cycles with a 4-mm radius of curvature.

The memristor has emerged as a promising candidate for the construction of a neuromorphic computing platform that is capable of solving the bottleneck of the von Neumann architecture. In terms of structure, memristors resembling biological synapses can emulate the various functions of the human brain, which is able to achieve large-scale neural networks as basic building blocks. Learning and information processing in neuromorphic computing utilizes the plasticity of synapses^4,6,7,10,12^. Synaptic plasticity is not always maintained synaptic structures, which can be strengthened or weakened. The process of changing the synaptic plasticity relates to learning and memorization. As shown in Fig. 5a, presynaptic neurons convert external stimuli to electrical signals and transmit them. Neurotransmitters in which chemical signals are secreted due to diffusion and the movement of ions such as Ca^2+^, K^+^, and Na^2+^ bind to receptors on postsynaptic neurons and then transmit signals. Because the characteristics of biological synapses might be mimicked by the conductance changes in memristive devices, here, we investigated the biomimetic properties of the agarose@AuNPs-based memristive device. The paired-pulse facilitation (PPF) in artificial synapses is important to quantify the dynamic filter of information transmission and spatiotemporal signal processing of the synapses, thereby mimicking a biological synapse. A paired spike (−1 V and 100 µs), which is determined from the results of Fig. 3a, was applied to the pre-synapse (Al top electrode) by using different interval ranges between 100 and 1000 µs. Then, the PPF index (A2/A1 × 100) was plotted versus the interval time between spikes, where A1 and A2 are the excitatory postsynaptic currents (EPSC) induced by the first pulse and the second one, respectively. The EPSC of the second pulse was larger than that of the first pulse. As shown in Fig. 5b, the PPF index increased as the time interval between spikes decreased. In addition, the PPF index could be fitted using a double-exponential decay function (red line), and the relaxation time constants τ_1_ and τ_2_ were found to be 11 μs and 519 μs, respectively, from the fitting result. This PPF function of the device can be understood based on the resistance change mechanism. As a sufficient electric field, which is caused by the applied spike, is formed between both electrodes, the number of free carriers in the active layer increases, and a gradual change in conductance can occur due to the strong trapping effect of the AuNPs and subsequent secondary spikes. On the basis of the PPF test results, we investigated the biomimetic properties of the agarose@AuNPs-based memristive device, and the results are shown in Fig. 5c. When 100 programmed paired spikes (−1 V-training spikes with 100 µs, and 0.1 V read voltage) were applied to the device to investigate its long-term potentiation (LTP) behavior, the conductance increased from 0.795 to 1.084 μS. The increased conductance caused by the potentiation process was returned to 0.795 μS after a consecutive depression process (2.0-V training spikes with 100 µs). Further, the energy consumption during the consecutive potentiation and depression of the device was calculated using following formulation:

3 $$ E = V_{spike\;amplitude} \times I \times t_{spike \;width} , $$

where V_spike amplitude_ is the amplitude of the applied spike, *I* is the the value of the current by a applied spike, and t_spikewidth_ is the duration of V_spike_. The average energy consumptions of the potentiation and the depression processes are 64.4 pJ/event and 164.4 pJ/event, respectively, as shown in Fig. 5d. One of the outstanding advantages of our memristive device is the possibility of applying it to flexible/wearable devices. For that reason, we prepared a memristive structure on an ITO/polyethylene glycol naphthalate (ITO-PEN) substrate and examined its deformation-dependent potentiation and depression behaviors of the device when it was bent from a flat state to a bent state with a 4-mm bending radius for up to 5000 bending cycles; the results are shown in Fig. 5e. The potentiation and the depression results were stable without any notable degradation due to the mechanical stress. This remarkable stability under mechanical stress is related to the physical interactions mediated by forces like those due to the hydrogen bonding between agarose chains^29,32,32^.

### Learning and inference capabilities by MNIST simulation

**Figure 6:**
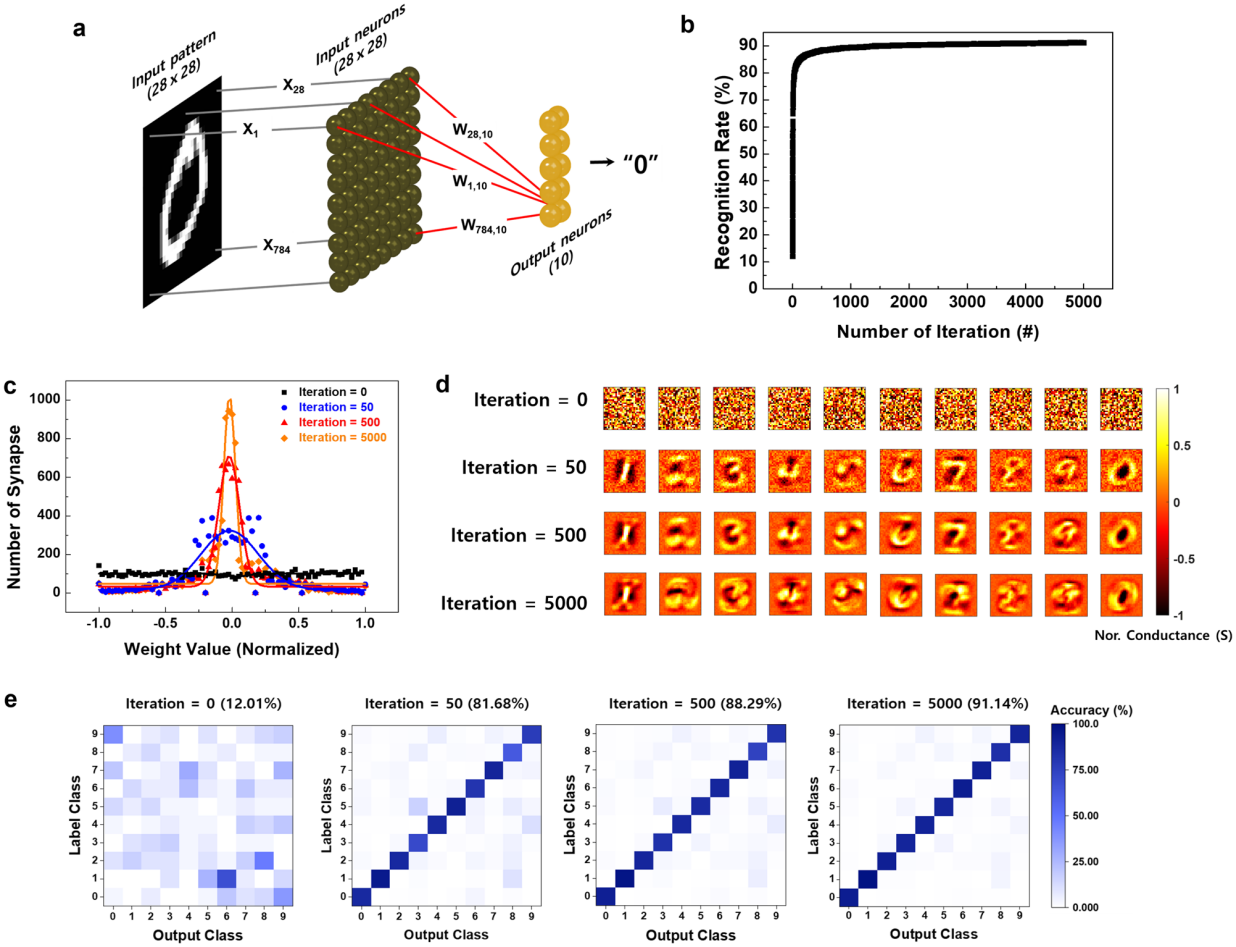
(**a**) Schematic diagram of a single layer network for the recognition process. The input pattern “0” (28 × 28), input neurons (28 × 28), and output neurons (2 × 5) are fully connected. (**b**) Recognition accuracy as a function of the number of training iterations. (**c**) Weight distribution at the 50th, 500th, and 5000th training iteration. (**d**) Weight-mapping images at the 0th, 50th, 500th, and 5000th training iterations. (**e**) Classifications for the inference test as a function of the number of training iterations.

Finally, to demonstrate the capability of learning and image recognition, we performed a Modified National Institute of Standards and Technology (MNIST) simulation on the basis of the potentiation and the depression characteristics of our synaptic device (Fig. 5c). The synaptic properties of our device were normalized before conducting MNIST simulation (Fig. S3). The details of the simulation are presented in the Supplementary Information. Figure 6a shows the pattern classification process with 784 input patterns (28 × 28 input pixels) fully connected to 784 input neurons (28 × 28 input neurons) and 10 output neurons (2 × 5 output neurons). Thus, 7840 pairs of synapses have their synaptic weight. Figure 6b shows the recognition accuracy without any training was 12%, but the recognition rate was improved to 81% after the 50th training. When the 500th and 5000th trainings were performed, the recognition rates were 88% and 91%, respectively. As the number of training iterations was increased, as shown in Fig. 6c, the distribution of the weight value became narrower. Figure 6d exhibits the mapping images from "0" to "9" after the 0th, 50th, 500th, and 5000th training iteration. The mapping images were recognized more clearly as the training was repeated. An inference test between label class and output class was performed using 60,000 and 10,000 different MNIST patterns, and the result could be visualized concretely by using the confusion matrix (10 × 10). The tiles in the initially chaotic confusion matrix were arranged diagonally with maximum saturation color after the 5000th training iteration (Fig. 6e).

## Conclusion

In summary, we demonstrated the memory and the synaptic performances of a biocompatible memristive device based on an agarose@AuNP nanocomposite layer. The Al/agarose@AuNP film/ITO memristive device exhibited reliable resistive switching characteristics (SET: −0.84 ± 0.21 V and RESET: 1.95 ± 0.25 V) with a large on/off ratio of 10^4^, and excellent retention for 10^5^ s under 80 °C. Compared to the device without AuNPs in agarose, the resistive switching behavior of the nanocomposite-based device was shown to be improved, which was ascribed to the trapping effect of the AuNPs. Furthermore, the agarose@AuNPs-based memristive device was demonstrated to be able to emulate biological synaptic functions through a changeable analog conductance behavior with low average energy consumption for the potentiation (64.4 pJ/event) and the depression (164.4 pJ/event) processes. In addition, the synaptic characteristics of our devices were maintained even when the devices were bent for repetitive 5000 cycles with a 4-mm radius of curvature. In the MNIST simulation based on actual device performance, a recognition rate of about 91% and accurate inference of digit classification were achieved. We believe that this work will provide a potential physical platform for biocompatible, wearable neuromorphic computing devices.

## Experimental details

Two wt% of agarose powder (DUKSAN) was added to deionized (DI) water, and the mixed solution was stirred at 80ºC for 24 h. Then, AuNPs (5 nm in diameter, Sigma-Aldrich) were mixed with the resulting solution in a volume ratio of 20%, followed by continuous stirring at 25ºC for 30 min. Indium-tin-oxide-coated polyethylene glycol naphthalate (ITO-PEN) substrates were sequentially cleaned by using sonication with acetone, methanol, and deionized water for 20 min each. Then, the substrates were dried under blowing N_2_ gas with a purity of 99.9%. An agarose@AuNP composite layer was spin-coated on the cleaned ITO-PEN substrate at 300 rpm for 5 s, 3000 rpm for 60 s, and 300 rpm for 5 s. The layer was baked on a hotplate at 120ºC for 60 min to remove the residual solvent and to stabilize the composite layer. Finally, the top Al electrodes (Al TE), 1 mm in diameter and 150 nm in thickness, were deposited on the composite layer by using thermal evaporation at a chamber pressure of 10^–6^ Torr.

The structural properties of the Al/agarose@AuNP composite layer/ITO-PEN memristive device were characterized by using scanning electron microscopy (SEM, Nova Nano SEM 230), Fourier-transform infrared spectroscopy (FR-IR, NICOLET iS50), and ultraviolet–visible spectroscopy (UV–VIS, LAMBDA 650S). The electrical properties of the composite-based memristive device were measured using a semiconductor parameter analyzer (Keithley 4200-SCS) coupled to a source meter (Keithley 2400).

## Supplementary Information

Supplementary Information.

## Data Availability

All data generated or analyzed during this study are included in this published article and its Supplementary Information files.
