# Supplementary material for: Biocompatible memristive device based on an agarose@gold nanoparticle-nanocomposite layer obtained from nature for neuromorphic computing
Source: Sci Rep. 2023 Apr 20;13:6491. doi: 10.1038/s41598-023-32860-6 (PMC10119280; doi:10.1038/s41598-023-32860-6)
Supplement: Supplementary file 1 — Supplementary Information. [file 41598_2023_32860_MOESM1_ESM.docx]

Supplementary Information

Biocompatible memristive device based on agarose obtained from nature for neuromorphic computing

Youngjin Kim, Jun Seop An, Donghee Lee, Seong Yeon Ryu, Yoon-Chul Hwang, Dae Hun Kim and Tae Whan Kim^*^

Department of Electronic Engineering, Hanyang University, Seoul, 04763, Republic of Korea

^*^Corresponding authors: [twk@hanyang.ac.kr](mailto:twk@hanyang.ac.kr) (T.W. Kim)

**
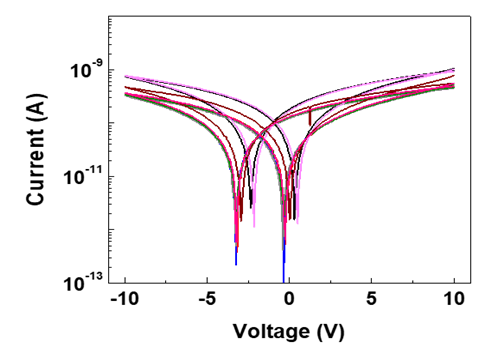
**

**Figure S1.** *I-V* characteristics of the Al/agarose/ITO-structured device for demonstrating the effect of including the AuNPs in the active layer.

**MNIST simulation**

**Fig. S2** shows the overall simulation as a simple flowchart. To calculate the weights to be mapped to the synaptic device, we used the MatlabR2018b tool to simulate a neural network. The neural network consists of an input layer with 784 neurons and an output layer with 10 neurons. The input and the output neurons are fully connected through 7,840 synapses. The rectified linear unit (ReLU) function for the activation function, the cross-entropy loss function for the error function, and the softmax function for processing the output signal are used. Each function is calculated as follows:

ReLU function: $y=\left\{ \begin{aligned} x, &x\geq0 \\ 0, &x<0 \end{aligned} \right.$ ,

Softmax function: ${p(y)}_{j}=\frac{e^{y_{j}}}{\sum_{k=1}^{K} e^{y_{k}}} for j=1, \cdots, K$ ,

Cross-Entropy loss function: $CE=-\sum_{i=1}^{10} t_{i}\log(p_{i})$ .

Here, $x$ is the sum of the input value multiplied by the weight of the synapse. MNIST patterns, 60,000 with 28$\times$28 pixels, are applied as input values through input neurons. We update the weight using gradient descent back-propagation as a learning algorithm. After training for 5000 iterations with this condition, the recognition rate of 10,000 MNIST inference patterns was 91.8% when the learning rate was 0.0002. The trained weight value is normalized and quantized according to a conductance behavior characteristic, such as the ratio of $G_{max}$/$G_{min}$, linearity, and multi-level status, and is mapped to the synaptic device. However, while the range of numerical trained weight values spans from negative to positive values, the range of real device-based weight values is limited to only positive ones. Therefore, two types of synaptic devices are required, an excitatory device ($G_{i,j}^{+}$) and an inhibitory device ($G_{i,j}^{-}$), to represent positive and negative weight values. Here, two types of synaptic devices are required, an excitatory device ($G_{i,j}^{+}$) and an inhibitory device ($G_{i,j}^{-}$), to represent positive and negative weight values. Therefore, one synapse unit is composed of each type of device, and the weight value is $G_{i,j}^{+}$+$G_{i,j}^{-}$. For hardware implementations, the model of integrate-and-firing (I&F) neurons based on capacitors is simulated. MNIST inference patterns are coded at a right-justified rate coding to convert the grayscale of pixels into the input spike signals and applied to the neural network for inference, as shown in **Fig. S4**.


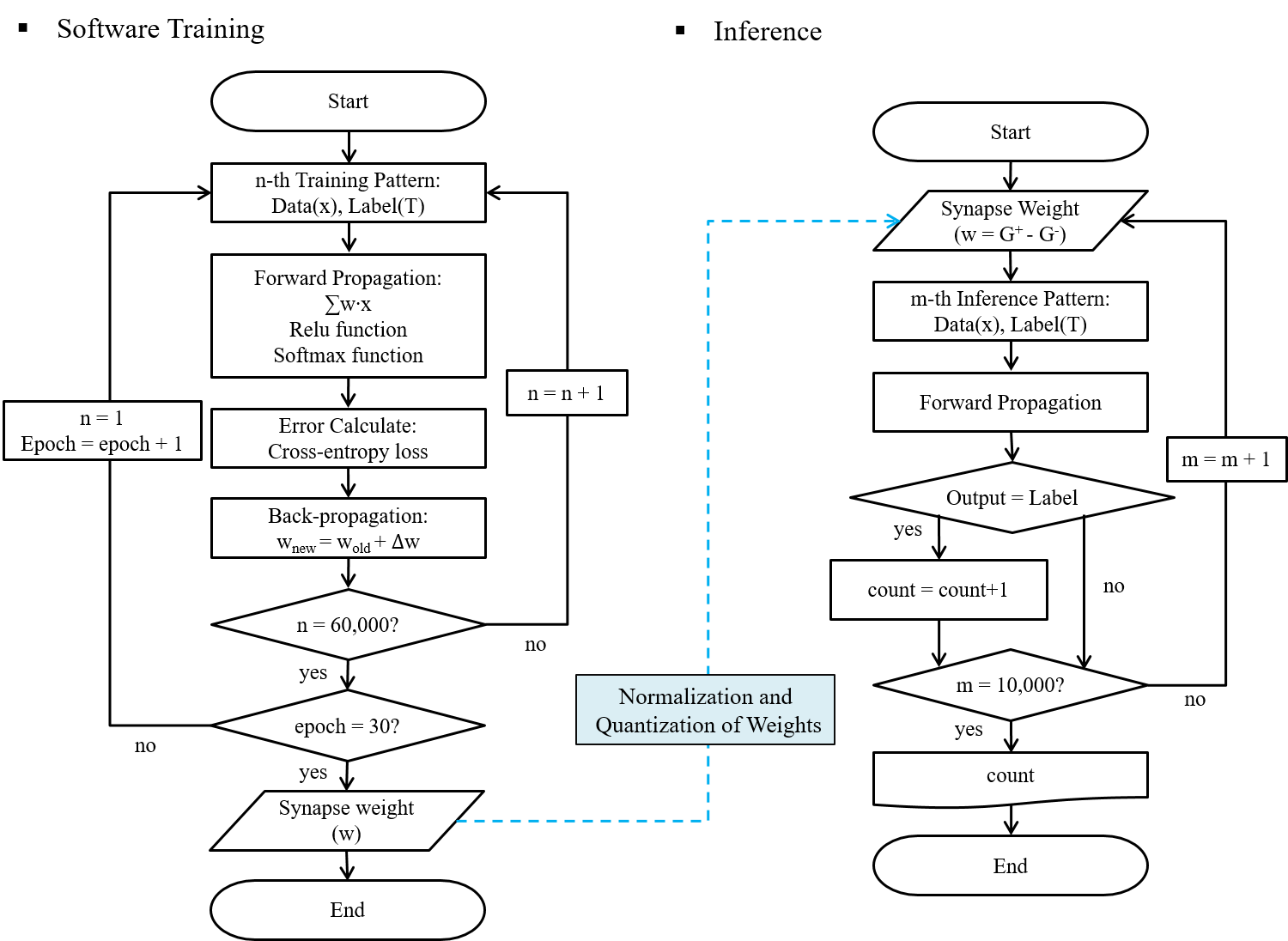


**Figure S2.** Flowchart for the learning and inference process.


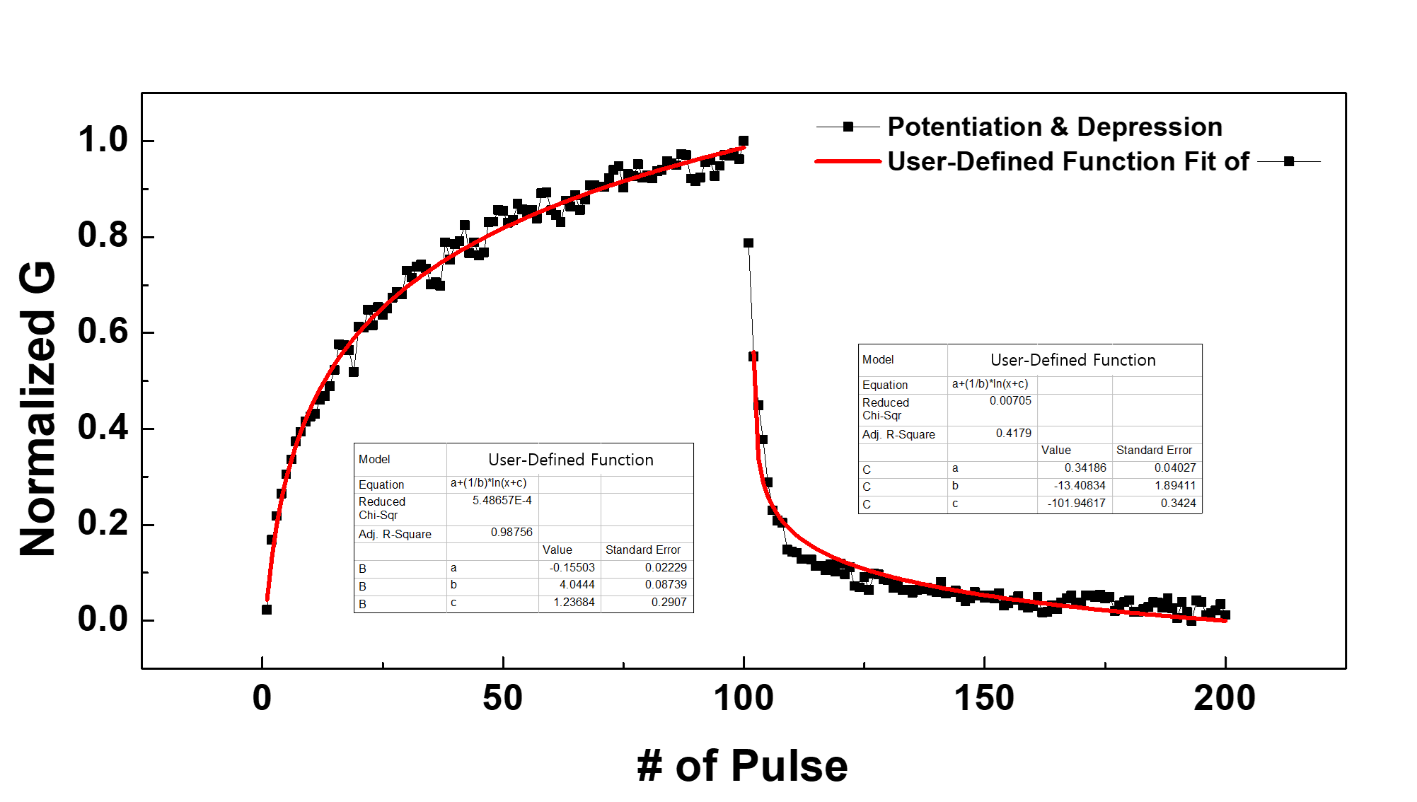


**Figure. S3.** Normalized conductance based on the potentiation/depression characteristics for the MNIST simulation.


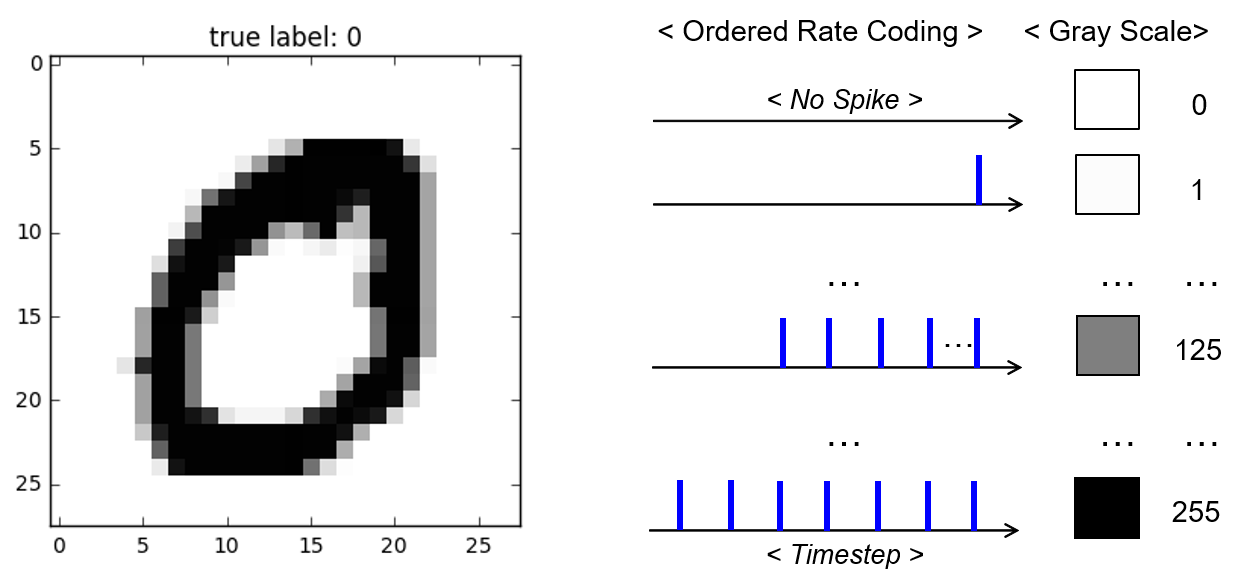


**Figure S4.** Schematic illustration of the righted-justified rate coding in MINST simulation.
